# Heat Treatment of Reishi Medicinal Mushroom (*Ganoderma lingzhi*) Basidiocarp Enhanced Its β-glucan Solubility, Antioxidant Capacity and Lactogenic Properties

**DOI:** 10.3390/foods10092015

**Published:** 2021-08-27

**Authors:** Attila Kiss, Petra Grünvald, Márta Ladányi, Viktor Papp, István Papp, Erzsébet Némedi, Iman Mirmazloum

**Affiliations:** 1 Agro-Food Science Techtransfer and Innovation Centre, Faculty for Agro-, Food- and Environmental Science, Debrecen University, 4032 Debrecen, Hungary; attilakiss.eger@gmail.com; 2 Department of Farm Animal Nutrition, Institute of Animal Physiology and Animal Nutrition, Hungarian University of Agriculture and Life Sciences, 7400 Kaposvár, Hungary; Grunvald.Petra@uni-mate.hu; 3 Department of Applied Statistics, Institute of Mathematics and Basic Science, Hungarian University of Agriculture and Life Sciences, Villányi Str. 29-43, 1118 Budapest, Hungary; ladanyi.marta@uni-mate.hu; 4 Department of Botany, Institute of Agronomy, Hungarian University of Agriculture and Life Sciences, Ménesi Str. 44, 1118 Budapest, Hungary; papp.viktor@uni-mate.hu; 5 Department of Plant Physiology and Plant Ecology, Institute of Agronomy, Hungarian University of Agriculture and Life Sciences, Ménesi Str. 44, 1118 Budapest, Hungary; papp.istvan@uni-mate.hu; 6 Expedit Nodum Ltd., 1162 Budapest, Hungary; expeditnodum@expeditnodum.com

**Keywords:** *G. lingzhi*, Reishi mushroom, beta-glucan, *L. casei*, prebiotic, antimicrobial

## Abstract

The effect of heat treatment on dried fruiting bodies of Reishi medicinal mushroom (*Ganoderma lingzhi*) is investigated. Control and samples treated for 20 min at temperatures of 70, 120, 150 and 180 °C were subjected for their free radical scavenging capacity, different glucans and total phenolic content determination. The growth rate of *Escherichia coli* and *Lactobacillus casei* supplemented with control and heat-treated samples is also investigated. The roasted mushroom samples at 150 °C and 180 °C showed the highest level of β-glucan (37.82%) and free radical scavenging capacity on 2,2-diphenyl-1-picrylhidrazyl (DPPH•) and 2,2-azino-bis-3-ethylbenzothiazoline-6-sulfonic acid (ABTS•+). The content of total phenolics (TPC) was also influenced by heat treatment and significantly higher TPC values were recorded in samples treated at 120 °C and 150 °C. The presence of reducing sugars was only detected after heat treatment at 150 °C (0.23%) and at 180 °C (0.57%). The heat treatments at 120, 150 and 180 °C, significantly attenuated the number of colony-forming units (CFU) of pathogenic *E. coli*, in a linear relationship with an elevated temperature. The supplementation of heat-treated Reishi mushroom at 120 °C resulted in the highest growth rate of probiotic *L. casei*. The obtained results in this study revealed the significant effect of short-term heat treatment by enhancing the antioxidant capacity, β-glucan solubility and prebiotic property of the dried basidiocarp of Reishi mushroom.

## 1. Introduction

Heat treatment of food has been extensively utilized to destroy microorganisms and stop enzyme reactions. However, such heat-treated foodstuffs exhibit definite diminishment in their nutritional values as a consequence of the loss of certain heat-sensitive components [1]. On the contrary, some recent studies have reported a significant increase in phenolic compounds of thermally processed foods [2,3,4].

It is well known that several food components of high nutritional value might be subjected to a significant loss as a consequence of thermal processing. It might be explained by the vulnerability of most of the bioactive compounds which are very frequently fairly unstable to heat. In some cases, however, thermal treatment does not result in an adverse change in the quality or composition of the studied foodstuff; conversely, improved traits can be experienced when heat treated. During thermal treatment, the formation of novel compounds of a marked antioxidant property, such as Maillard reaction products (MRP), can be observed. Therefore, the loss of some of the naturally occurring antioxidants or heat-liable nutrients can be counterweighted by the formation of new antioxidants leading to an enhanced overall antioxidant activity of the food undergoing heat treatment. As a result, a final product of higher biological and nutrition values is yielded being associated with definite beneficial health effects such as the MRP from heated histidine and glucose showing peroxyl radical scavenging activity, an indicator of antioxidative activity in vitro [5].

*Ganoderma* (*Polyporales*, *Basidiomycota*) is a large and economically important cosmopolitan polypore genus with about 180 white-rot fungi species [6]. The most significant species of the genus is the widely cultivated *G. lingzhi* (*G. lucidum* s. auct.), which has been used in traditional Chinese medicine for centuries. Due to the woody fruiting body, and the characteristic bitter taste of many *Ganoderma* species, they are classified as nonedible mushrooms [7], but the presence of several health-promoting and biologically active molecules in *Ganoderma* species justified well its application in functional foods and supplements [8,9]. Several bioactive molecules such as polysaccharides (mainly β-glucans), terpenoids, phenolic compounds, steroids, saturated, monounsaturated and polyunsaturated fatty acids, vitamins (B1, B2, B6), proteins and minerals with established health benefits have been reported from *G. lingzhi* [10,11,12]. The anti-obesity, anti-inflammatory and prebiotic properties of *G. lingzhi* have been documented in several reports and scientific reviews [13,14,15,16]. The non-digestible and prebiotic β-glucans are the main constituents of mushroom polysaccharides that can reach the large intestine to enhance the microbiota [16] and, therefore, can be considered as a functional or nutraceutical ingredient [17].

Many studies have been engaged in the assessment of the antioxidant activity, including the radical scavenging activity, reducing power, and antioxidant enzyme activity, subsequent to heat treatment. It was pointed out that the thermally processed products displayed elevated chain-breaking and oxygen-scavenging activities [18,19]. Previous studies have reported that the antioxidant properties of garlic [20] and tomato [21] were enhanced as a consequence of thermal treatment due to the disruption of the cell wall and liberation of phenolic compounds from their insoluble forms. The polyphenolic (both free and bound) flavonoids contents and antioxidant activities of Shiitake mushroom showed a significant increase in mushroom extract subsequent to heat treatment of raw materials at 100 and 121 °C for 15 or 30 min [22]. The antioxidant and physicochemical properties of oat grains studied after sand roasting at 280 °C for 15 s have been reported with a significant enhancement [23]. As a result of roasting, a significant increase was observed in the reducing power and the DPPH radical scavenging activity of the roasted oat groats. Similar findings were reported on rice hull where the content of free phenolic acids increased as a result of heat treatment at 80 to 140 °C for 1 to 5 h [24].

Based on the abovementioned precedents, the objective of this study is to investigate the changes in the content of the phenolic compounds, antioxidant activity and β-glucan extractability of thermally processed Reishi mushroom fruiting body and to evaluate the prebiotic effect of the obtained samples on *Lactobacillus casei* growth.

## 2. Materials and Methods

### 2.1. Materials and Instruments

Folin–Ciocalteu’s phenol reagent, gallic acid and 1,1-diphenyl-2-picrylhydrazyl (DPPH) were purchased from Sigma Chemicals (St. Louis, MO, USA). Mushroom β-glucan quantification kit was obtained from Megazyme Int. (Wicklow, Ireland). All of the chemicals and solvents were of analytical grade. *L. casei* (ATCC 334, KWIK-STICK) and *E. coli* (ATCC 25922, MECCOUNTI) were used in this study. The spectrophotometric measurements were performed using an 1800 UV–VIS spectrophotometer (SHIMADZU Inc., Kyoto, Japan) and a microplate reader (PowerWave XS2, BioTek, Winooski, VT, USA).

### 2.2. DNA Molecular Marker and Identification of Reishi Mushroom

The applied basidiocarp of Reishi mushroom was a commercially produced product of Oriens Gomba Ltd., Pest, Hungary. The specimen (Oriens-1) was systematically identified by means of genetic marker sequencing. A Phire^®^ Plant Direct PCR Kit (Thermo Scientific, Waltham, MA, USA) was used to generate molecular data. Using the primers ITS1F (5′-CTTGGTCATTTAGAGGAAGTAA-3′) and ITS4 (5′-TCCTCCGCTTATTGATATGC-3′) [25] and the PCR protocol [26], the ITS (internal transcribed spacer) regions of the nuclear ribosomal DNA were amplified. The PCR products were visualized and checked by gel electrophoresis and the purified PCR products were sequenced using the same primers at the Biological Research Centre (Szeged, Hungary). The nucleotides chromatogram was checked, assembled and edited with CodonCode Aligner 7.0.1. The obtained nucleotide sequence amplified from the applied Reishi mushroom in this study was named (Oriens-1) and deposited in the NCBI GenBank (www.ncbi.nlm.nih.gov/genbank/ accessed on 23 August 2021) under the accession number of MW139644. The phylogenetic analysis based on the ITS sequences of Oriens-1 and of a selection of reference sequences from other laccate *Ganoderma* species was also conducted to show the position of the studied mushroom strain.

### 2.3. Heat Treatment and Extraction

The dried basidiomata of Reishi mushrooms were grinded using a coffee grinder. The obtained fluffy basidiocarp tissues were defatted with petroleum ether and re-dried at 35 °C in a hot air oven (Memmert, ULE500, Schwabach, Germany) for 16 h before the heat treatment (roasting) to equally minimize the moisture content of all samples. From the dried basidiocarp, 10 g was distributed in different round glass containers (25 cm ↔ × 5 cm↑) and placed in a hot air oven where different temperatures (70, 120, 150 and 180 °C) were applied for 20 min for each set of dried materials. The samples were then cooled down to room temperature and stored in 1 L Duran glass bottles with airtight caps until extraction. A sample without any heat treatment was considered as control.

The extraction was performed as described earlier [27]. Briefly, the differentially heat-treated samples were mixed with 75% ethanol (1:10 *w*/*w*) and placed in an ultrasonic bath for 15 min at 35 °C in 1 L DURAN bottles prior to extraction at 40 °C for 8 h in an orbital shaking water bath. The extracts were filtered (Whatman No. 4 paper filters) and concentrated to one-fourth of the original volume in a vacuum rotary evaporator. Subsequently, ice-cold ethanol (five times the remaining volume) was added to the flask and kept at 4 °C for 24 h. The precipitate was collected after centrifugation, kept at −80 °C for 2 h and lyophilized in a Christ ALPHA 2–4 LSC freeze dryer (Martin Christ Gefriertrocknungsanlagen GmbH, Osterode am Harz, Germany) at −40 °C to −45 °C for 48 h at 5 Pa, to obtain a light brown extract that was stored at room temperature until use.

### 2.4. Determination of Total Phenolics Content (TPC)

The Folin–Ciocalteu method with modifications was applied to estimate the total phenolics content of the *G. lingzhi* basidiocarp extracts [28]. An amount of 100 µL of each sample extract (solution prepared in 1:1 ratio *w*/*w* of lyophilized extract and deionized water) was mixed with 500 µL of deionized water and 100 µL of 10% Folin–Ciocalteu reagent. The mixture solution was gently mixed and incubated for 6 min at room temperature in the dark; when 1 mL of 7.5% (*w*/*v*) Na_2_CO_3_ was added to the reaction and after another 60 min of incubation at dark, the absorbance was recorded at 760 nm. Gallic acid (0.156–5 mg mL^−1^) was used to construct a calibration curve ($R^{2}\;$= 0.9934), and the TPC was calculated as gallic acid equivalents in mg GAE g^−1^ DW of dried mushroom samples (control and heat-treated). The assay was conducted in triplicate, and the results are presented as mean ± standard deviations.

### 2.5. Free Radical-Scavenging Activity (DPPH and ABTS Assay)

The method of Brand-Williams et al. [29] was applied with small modifications. The reaction consisted of 1.9 mL of 130 μM DPPH (completely dissolved in absolute ethanol) plus 0.1 mL of different Reishi mushroom extracts (solution prepared in 1:1 ratio *w*/*w* of lyophilized extract and deionized water). The mixtures were incubated at 23 °C for 20 min in dark. The radical-scavenging activity was expressed as the percentage of DPPH decolourization (determined at 517 nm) using the following Equation (1):

% Radical Scavenging Activity = ([*A*_DPPH_ − *A*_S_]/*A*_DPPH_) × 100 (1)

where *A*_DPPH_ is the absorbance of the reaction mixture containing 0.1 mL of H_2_O and *A*_S_ is the absorbance of the solution containing 0.1 mL of each sample after 20 min. The radical scavenging activity was expressed as the percent of DPPH inhibition and the results presented as the mean ± standard deviations.

For the ABTS spectrophotometric method, the working solution consisted of 2,2′-Azino-bis(3-ethylbenzothiazoline-6-sulfonic acid) diammonium salt (7 mM) and potassium persulfate (2.45 mM) in deionized water prepared 16 h before the assay measurement (kept at dark and room temperature) to form ABTS•+. The solution was then diluted with deionized water to obtain an absorbance of 1.5 at 420 nm. From this working solution, 250 µL was transferred to different wells of a 96-well flat-bottom plat and using an 8-channel pipette 50 µL (5 × dilution) of control and heat-treated Reishi extracts was added to each well at the same time. The changes in absorbance were recorded in microplate spectrophotometer (PowerWave XS2, BioTek, Winooski, VT, USA). Trolox (Sigma Chemical Co., St. Louis, MO, USA) was used to obtain the standard curve. The Trolox equivalent antioxidant capacity (TEAC) was calculated using following Equation (2):

TEAC (mg Trolox eq) = (∆*A*_sample_)/(∆*A*_Trolox_) × (TC/SC) (2)

where ∆*A*_(sample)_ is the change of absorbance when extracts were added and ∆*A*_(Trolox)_ is the change in absorbance when Trolox standard solution was added; TC shows the concentration (mg/mL) of the Trolox standard and SC (Sample concentration) shows the concentration of the Reishi sample (mg/mL). All samples were analysed in triplicate.

### 2.6. Measurement of α-glucan and Reducing Sugars

To determine the α-glucan contents of the heat-treated samples, 100 mg of original basidiocarp was added to Fisherbrand glass culture tubes. To dissolve the starch/glycogen, 2.0 mL of ice-cold KOH (2 M) was added to each tube and mixed vigorously. After 20 min of incubation and regular vortexing in an ice water bath, 7.0 mL of 1.2 M sodium acetate buffer (pH 3.8) was added to each tube and mixed well. A total of 0.2 mL of amyloglucosidase (1630 U/mL) plus invertase (500 U/mL) was mixed with the tube content and incubated at 40 °C for 30 min in a water bath with intermittent mixing on a vortex stirrer. Aliquot of 1.0 mL of the solution (from 9.3 mL final volume) was taken and centrifuged at 400 RCF for 10 min from which, eventually, 0.1 mL of the supernatant was transferred to glass test tubes (16 × 100 mm) and incubated at 40 °C for 20 min with the addition of 0.1 mL of sodium acetate buffer (200 mM, pH 5.0) plus 3.0 mL of GOPOD Reagent. The absorbance of these solutions was measured at 510 nm against the blank reagent composed of 0.2 mL of sodium acetate buffer (200 mM, pH 5.0) plus 3.0 mL of GOPOD Reagent in a microplate spectrophotometer (PowerWave XS2, BioTek, Winooski, VT, USA).

The glucose content (total glucan plus free glucose and glucose from sucrose) was analysed by measuring the absorbance at 510 nm against the blank reagent composed of 0.2 mL of sodium acetate buffer (200 mM, pH 5.0) plus 3.0 mL of glucose oxidase–peroxidase reagent (GOPOD).

The reducing sugars were quantified using the Luff-Schoorl Titration method for 2.5 g of each heat-treated and control samples as recommended by the Official Journal of the European Union (COMMISSION REGULATION (EC) No 152/2009), and the results were expressed as g 100 g^−1^ of dry materials presented as mean values with standard deviations.

### 2.7. Measurement of Total and β-glucan Content

The β-(1,3)-(1,6) d-glucan content of the samples was detected by following the manual of Megazyme test kit in triplicates with minor modifications. The dissolution and dispersion of the heat-treated samples was achieved in ice-cold sulfuric acid (12 M) with vigorous mixing (10–15 s) every 15 min in an ice water bath for 2 h by sequentially adding deionized water to each tube while mixing and vortexing (10–15 s). The tubes with loosened caps were placed in a hot water bath (~100 °C) and after 5 min, the caps were tightened for 2 h of incubation at 100 °C. To neutralize the solution, 6.0 mL of KOH (10 M) was then added and mixed well with the tube contents. The contents of each tube were transferred quantitatively to volumetric flasks and the volume was adjusted to 100 mL with 200 mM sodium acetate buffer (pH 5). Aliquots (2 mL) of the solution were centrifuged at 2700 RCF for 10 min and 0.1 mL of the supernatant was incubated with 0.1 mL of a mixture of exo-1,3-beta-glucanase (20 U/mL) and beta-glucosidase (4 U/mL) in 200 mM sodium acetate buffer (pH 5) at 40 °C for 60 min. The glucose oxidase–peroxidase reagent (3.0 mL) was then added to each reaction tube and incubated at 40 °C for another 20 min. The β-glucan content was determined by subtracting the α-glucan content from the total glucan content.

### 2.8. Bacterial Cell Culture and Growth Rate Determination

An inoculating loop of *L. casei* (ATCC 334, KWIK-STICK) and *E. coli* (ATCC 25922, MECCOUNTI) was suspended in phosphate buffer followed by homogenisation/resuscitation for an hour. The solution was analysed with a spectrophotometer at 600 nm (OD600) to ensure the cell density reached about cell populations of 10^8^–10^9^ cells mL^−1^. One mL of the above mentioned bacterial suspension was added to 9 mL of milk (Pilos UHT, 1.5%, sterilized with filtration before use) when 0.3 g of different Reishi mushroom samples was added into each solution. The mixtures were incubated at room temperature for an hour with gentle vortexing every 10 min, of which 100 µL was taken and added into 5 mL MRS (VWR) for *L. casei* and TBX (VWR) for *E. coli* in Fisherbrand culture tubes. The milky MRS and TBX without Reishi mushroom were used as controls. The tubes were incubated at 37 °C for 72 h in CO_2_-generating sachets (Microbiology Anaerocult A, Merck KGaA, Germany) in an anaerobic culture jar system. The plate count method was used to monitor the growth rate of pathogenic *E. coli* and probiotic *L. casei* supplemented with differentially heat-treated Reishi samples. The samples of incubated bacterial cells were subjected for serial dilution (10^−1^–10^−5^) and colony-forming units (CFU) count analysis (100 µL) on MRS and TBX agar plates after aerobic and anaerobic incubation for 24 h (initial count) and 72 h (end count) at 37 °C. Blank MRS and TBX agar plates were considered as blank controls. The Frutafit^®^ HD Inulin oligofructose (average DP ranged between 9 and 12) from chicory root (SENSUS, Roosendaal, The Netherlands) was used as an external control with proven prebiotic property.

### 2.9. Statistics

The effect of heat treatment on reducing sugars was compared by Student’s *t*-test. The total phenolics content (TPC) and the antioxidant properties (DPPH, ABTS) were analysed by one-way ANOVA models. We performed one-way MANOVA tests to detect the effect of heat treatment on glucan contents and the lactogenic and antibacterial properties of heat-treated *G. lingzhi*. Significant overall MANOVA results were followed by one-way ANOVA tests for all the variables (α-glucan, β-glucan and total-glucan; *E. coli* and *L. casei* values in initial and end count) with Bonferroni’s correction. The time effect on *E. coli* and *L. casei* was compared by Student’s paired *t*-tests. The antioxidant capacity in the differentially heat-treated samples were checked for linear increase by calculating the significance of the trend slope. The required normality assumptions of the model residuals were accepted by their skewness and kurtosis (as their absolute values were all below 1) and homogeneity assumptions were tested by Levene’s test. In case of significant results, Games-Howell’s post hoc tests were applied, in other cases, Tukey’s tests were used for the pairwise comparisons. Finally, principle component analysis was performed to visualize the relationship among the measured variables and the samples using a bi-plot.

## 3. Results and Discussion

### 3.1. Taxonomical Identification of Reishi Mushroom

**Figure 1:**
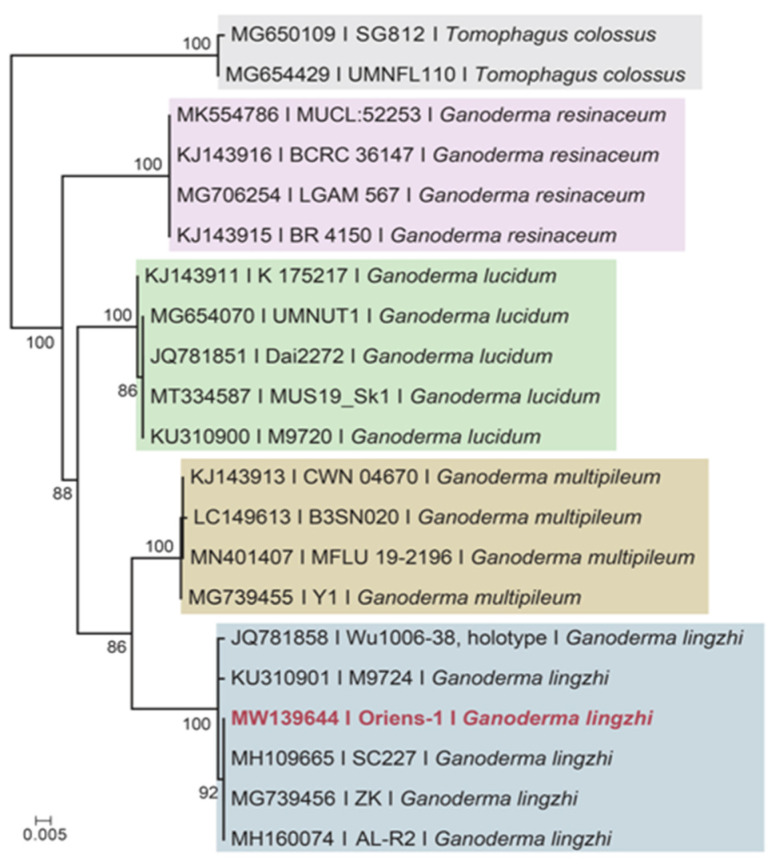
Maximum likelihood (RAxML) tree of ITS of the studied material (marked in red) and related specimens. *Tomophagus colossus* was served as outgroup. The scale bar indicates 0.005 expected changes per site per branch.

Due to the diverse inter and intraspecific phytochemical variability of Reishi mushrooms amongst the literatures [30], the molecular identification of the applied specimen was conducted to compare the results of our study with other published reports on Reishi or *Ganoderma*. The sequence of ITS nuclear ribosomal DNA from the applied mushroom was clustered with *G. lingzhi* sequences (incl. the holotype) in the presented phylogenetic tree (Figure 1) in a well-supported clade (MLBS (Maximum Likelihood Bootstrap Support values) = 100%). Although the content and properties of active molecules in Reishi mushroom samples can vary upon different harvesting times, drying temperatures and extraction methods [31], the proper identification of the species should be outlined and be considered for different experimental research and potential commercialization.

### 3.2. Effect of Heat Treatment on Reducing Sugars and Total Phenolics Content (TPC) of G. lingzhi

Reducing sugars were not detected in control and heat-treated samples at 70 °C and 120 °C. However, the higher temperature treatments (150 °C and 180 °C) resulted in the formation of reducing sugars of 0.23% and 0.56%, respectively (Table 1). The difference between the reduced sugar contents in the samples treated at 150 °C and 180 °C was significant (*t*(4) = 38.61; *p* < 0.001). There were different reported values with considerable variations for total and reducing sugar contents in Reishi mushrooms. This was mostly related to the origin of the studied strain, and many other associated factors during the growth to harvest period. The content of fructose and glucose in a Serbian strain of *G. lucidum* was around 5% and 1%, respectively, whereas in the same study, the authors reported no detectable fructose and glucose in their Chinese-originated specimen similar to our findings in the control samples [32]. In another report [33], *G. lucidum* from Northeast Portugal contained 1.2% of combined fructose and glucose which is another indication of sugar profile variability amongst the Reishi mushroom. The dry heat treatment at temperatures above 140 °C can give rise to the formation of reducing sugars upon starch and non-reducing sugars hydrolysis [34] as detected in our samples.

**Figure 2:**
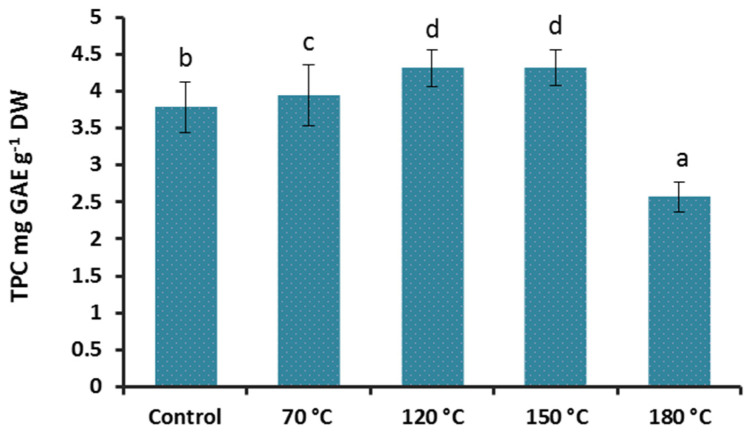
Total phenolics content (mean ± StDev) in Reishi mushroom (*G. lingzhi*) basidiocarp after heat treatment. Different letters are for statistically different values (Tukey’s post hoc test, *p* < 0.05).

The direct relationship between the antioxidant capacity and the polyphenols content has been well documented in several reports and is important when it comes to health-promoting properties of natural ingredients. To learn the effect of high temperature treatment on polyphenols in our samples, the gallic acid standard curve was used for quantification. The heat treatments significantly influenced the content of total phenolics in Reishi extracts (F(1;4) = 481.70; *p* < 0.001; Figure 2). The samples treated at 70, 120 and 150 °C showed a significantly higher TPC (3.94, 4.31, and 4.31 mg GAE g^−1^ DW) than the control (3.78, mg GAE g^−1^ DW). The 180 °C treatment reduced the TPC to 2.65 mg GAE g^−1^ DW significantly. Similar results were reported from heat-treated or roasted Shiitake mushroom in two independent studies where the heat treatment at 100 and 121°C for 13 and 30 min, and roasting at 80, 100 120,140 and 160 °C for 60 min, significantly increased the detected polyphenolic contents [22,35].

In contrast to the results of Hwang et al. [35], where the 180 °C treatment for half an hour had no negative impact on the TPC of the Shiitake mushroom, the same temperature treatment for 20 min reduced the TPC of our sample significantly when compared with the control.

### 3.3. Effect of Heat Treatment on Antioxidant Properties of G. lingzhi

**Figure 3:**
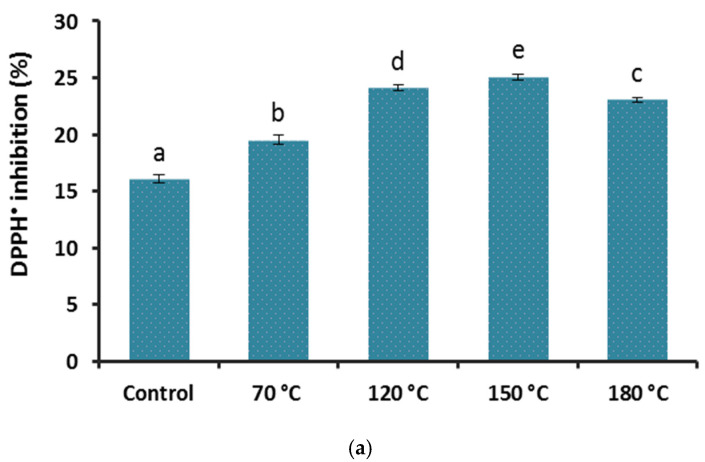

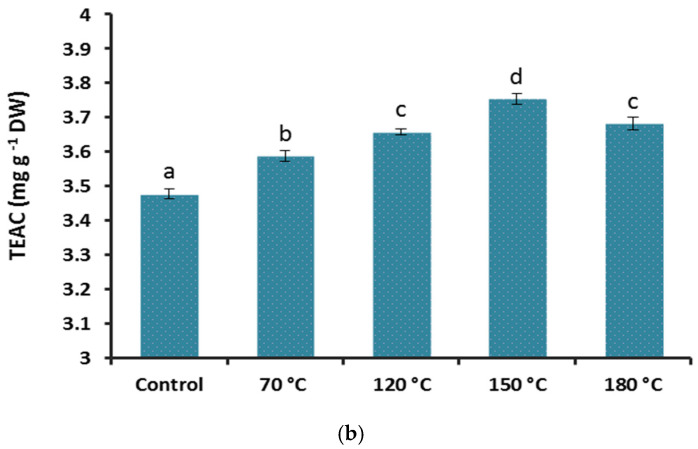
The antioxidant capacity (mean ± StDev) of (*G. lingzhi*) basidiocarp extract (2.5 mg mL^−1^) after heat treatment in DPPH (**a**) and ABTS (**b**) assays. Different letters are for statistically different values (Tukey’s post hoc test, *p* < 0.05).

The free radical scavenging power of all Reishi samples was significantly enhanced after the heat treatment when compared with the control in both DPPH (F(4;10) = 462.76; *p* < 0.001; Figure 3a) and ABTS (F(4;10) = 139.52; *p* < 0.001; Figure 3b). The antioxidant capacity in the differentially heat-treated samples showed a linear increase at higher temperatures up until the 150 °C heat treatment (slopes: 3.16 and 0.09 for DPPH and TEAC, respectively, all with *p* < 0.001) and declined at 180 °C in both assays. Even after the highest temperature treatment the antioxidant capacity was higher than the untreated samples. The values from our DPPH assay (16–25% of inhibition) were relatively lower than those reported from an Indian Reishi strain (IC_50_ = 0.29 mg mL^−1^) [36], but comparable to a report from a Chinese Reishi mushroom (IC_50_ = 3.66 mg mL^−1^) [37]. The lower antioxidant capacity in our extract can also be due the applied extraction method that was selected to obtain the polysaccharides along with the phenolics in which the organic soluble phenolic compounds were partially excluded when the lower phase of the extract was collected and processed to dryness.

Comparing the sharper declined TPC values than the antioxidants quantity at 180 °C treatment, it is evident that other compounds in our Reishi mushroom extract, such as reducing sugars that were only present at high-temperature-treated samples, were also contributing to the detected antioxidant capacity. Therefore, in the overall estimation of the heat treatment effect on antioxidant capacity, a final enhancement could be concluded at all of the heat-treated samples compared to the control.

### 3.4. Effect of Heat Treatment on Glucans Content of G. lingzhi

**Figure 4:**
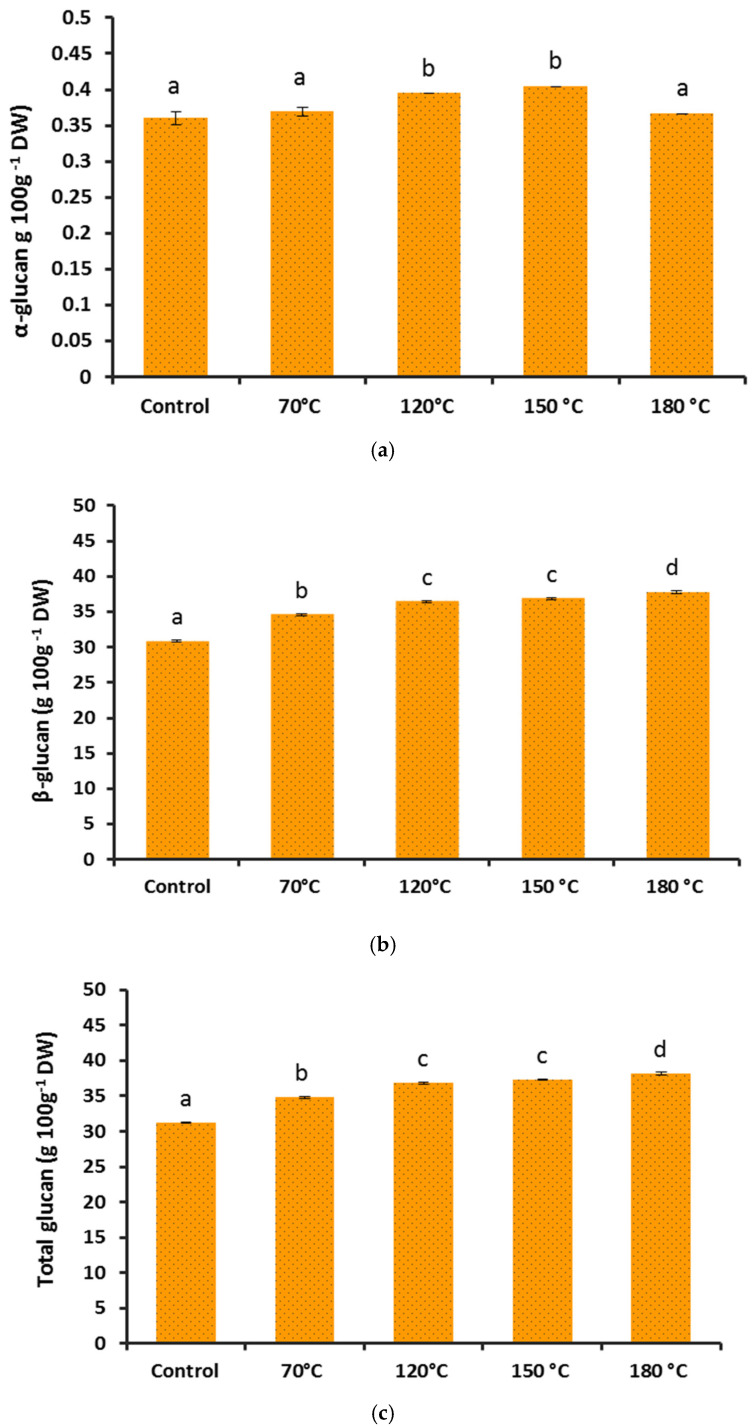
The effect of heat treatment on *G. lingzhi*’s basidiocarp α-glucan (**a**), β-glucan (**b**) and total glucan (**c**) contents (mean ± StDev). Different letters are for statistically different values (Games-Howell’s post hoc test, *p* < 0.05).

Polysaccharides such as β-glucans are one of the main constituents of mushroom cell walls and play an important role in adaptive immunity, therapeutic properties and the amelioration of overall wellbeing [12,38,39,40]. The extractability and content of α- and β-glucans of the applied Reishi mushroom were influenced by treatment significantly according to the overall MANOVA test (Wilk’s λ < 0.001; *p* < 0.001) that was detectable in each variable (α-glucans: F(4;10) = 33.75, β-glucans: F(4;10) = 747.70; total-glucans: F(4;10) = 786.97, all with *p* < 0.001). Except the insignificant changes from the control in the α-glucan content in samples treated at 70 °C and 180 °C, the other two treatments (120 °C and 150 °C) resulted in a significantly higher α-glucans content (Figure 4a).

The measured β-glucan contents in all of the heat-treated samples were significantly higher when compared with the control (Figure 4b). Almost a 7% increase in β-glucan content was detected in the samples treated for 20 min at 180 °C (37.82%) in comparison to the control (30.88%). A statistical analysis of the total glucans content also revealed significantly higher values in all of the heat-treated samples than the control (Figure 4c). The β-glucan contents reported from Reishi or Ganoderma mushrooms were in a wide range of variability from as low as 4 g 100 g^−1^ to as high as 55.9 g 100 g^−1^ of dried materials [41,42]. This high range of deviation in the mentioned values can reflect the taxonomical confusion, improper identification, different growth condition, cultivation practices and the applied extraction methods and quantification techniques.

The partial degradation and molar mass reduction, the structural conformation or polymerization and also changes in the intrinsic viscosity of β-glucans have been reported after thermal processing in cereals and mostly in aqueous solutions [43,44,45]. We also detected a slight but linear and temperature-dependent decrease (the lowest cP at 180 °C) in the viscosity of the heat-treated Reishi mushroom basidiocarp in water (data are not shown). Despite the potential molecular disintegration that may occur at extreme high temperature processing and, more reportedly, in solutions containing chelating ions or preservatives such as ascorbic acid, the overall outcomes of dry Reishi mushroom basidiocarp heat treatments on β-glucans were of significant improvement in this study.

### 3.5. Lactogenic and Antibacterial Properties of Heat-Treated G. lingzhi

The applied microbiological assay to estimate the effect of Reishi supplementation on the growth rate of the applied stains revealed the potential prebiotic effect of the studied mushroom that was confirmed by the highly significant MANOVA overall test (Wilk’s λ < 0.001; *p* < 0.001) together with the follow-up tests for the measured *E. coli and L. casei* initial and end count growth rates (F(5;12) > 8.97; *p* < 0.001).

The heat treatment reduced the number of culturable pathogenic *E. coli* in both initial and end-point counts significantly (*p* < 0.05; Table 2). The remarkable and strongest inhibitory effect of heat-treated Reishi mushroom was observed in the samples treated at 150 °C and 180 °C with a 1.8 and 4.33 Log CFU decrease, respectively, when compared to the control. The more pronounced inhibitory effect on *E. coli* growth was detected after 72 h of incubation confirming the treatment-dependent inhibition. Similar to the untreated Reishi sample, the Frutafit HD inulin did not show any inhibitory effect on *E. coli* growth and proliferation after 24 and 72 h of incubation.

The antibacterial effect of Reishi mushroom has been reported earlier for *B. cereus*, *P. aeruginosa*, *E. cloacae*, *S. typhimurium*, *S. aureus*, *M. flavus*, *L. monocytogenes* and *E. coli* [32]. Amongst the studied pathogenic strains, *E. coli* was the most resistant and its growth inhibition required 3 mg mL^−1^ of the applied mushroom extract. Our results on *E. coli* growth inhibition by heat-treated samples can be considered for further research to fully expose the potential antibacterial properties of heat-treated Reishi mushroom on different pathogenic strains.

The lactogenic effect of the applied Reishi mushroom improved significantly up to the level of Frutafit HD inulin (5.512 ± 0.08 Log CFU) and even higher after the heat treatment at 70, 120 and 150 °C and slightly decreased after 180 °C treatment that was still significantly higher than in the untreated sample (Table 2). The overall growth rate of *L. casei,* considering both the initial (5.594 ± 0.02 Log CFU) and end count (9.33 ± 0.03 Log CFU), was recorded for the sample treated at 120 °C for 20 min (Table 2). This can be correlated with the more β-glucan availability as a result of heat treatments presented above (3.4.).

Although the prebiotic effect of Reishi mushroom has been already established [27,46], according to our best knowledge, such a significant improvement on its prebiotic property enhancement has not been reported before.

### 3.6. Principal Component Analysis

**Figure 5:**
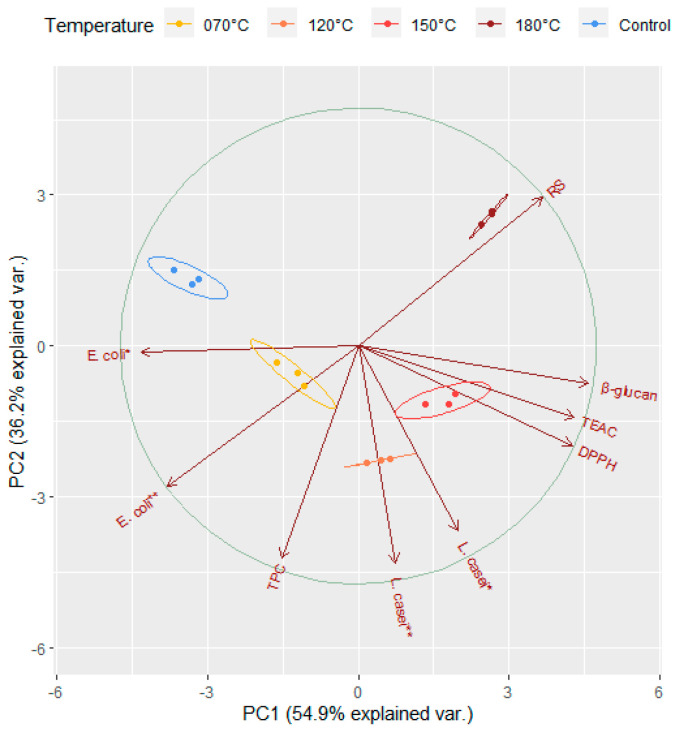
Principal component analysis (PCA) bi-plot for the observed variables. Vector captions: TEAC, DPPH, β-glucan, *E. coli* *, *E. coli* **, *L. casei* *, *L. casei* **, RS (Reducing Sugars Content) and TPC (Total Phenolics Content). Brown arrows represent the measured variables and points of different colours represent sampling points relative to the heat treatment completed with 99% confidence interval ellipses. *: initial Log CFU count; **: end Log CFU count.

To summarise the main points of the complex relationship of the examined variables under heat treatment, we performed a principal component analysis and visualised the results in a bi-plot (Figure 5) with two principal components (PC1 and PC2). The two principal components explained 91.1% of the total variation (54.9% and 36.2%, respectively). In the plot, the arrows represent the measured variables and the points of different colours represent the sampling points relative to the heat treatment. Sampling points on the same side as a given variable should be interpreted as having a high contribution to it. The magnitude of the arrows shows the strength of their contribution to each PC. Arrows pointing in similar or opposite directions indicate positively or negatively correlated variables, while those close to be perpendicular indicate a low or no correlation. The sizes of the coloured ellipses refer to a 99% probability level, and show a clear partitioning of the different heat treatments along the PC1, and the partition of control along PC2. PC1 was strongly and positively correlated to variables TEAC, DPPH and β-glucan, and negatively to *E. coli* *, *E. coli* ** and reducing sugars (RS). PC2 was strongly and negatively correlated to *E. coli* **, *L. casei* * and *L. casei* **, while positively to RS. TEAC, DPPH and β-glucan which were the highest at 150 °C, while *L. casei* *, *L. casei* ** and TPC were the highest at 120 °C or close to this temperature. *E. coli* * *and E. coli* ** were the highest at 70 °C while RS at the highest temperature (180 °C). Considering Figure 5, together with the Pearson correlation table (Table 3), we could detect the association directions and magnitudes among the variables. TEAC, DPPH, β-glucan and *L. casei* * were significantly, positively and pair wisely correlated, while *L. casei* * was significantly and positively correlated to *L. casei* **, too; that is, positively correlated to TPC. *E. coli* * and *E. coli* ** were significantly and negatively correlated to RS, TEAC, DPPH and β-glucan.

## 4. Conclusions

The low-cost and efficient heat treatment of Rishi dry basidiocarps at temperatures up to 150 °C for as short as 20 min resulted in a positive and significant enhancement in all of the studied parameters (the TPC, antioxidants capacity, β-glucan and *Lactobacillus casei* growth rate) with regard to health-promoting properties. A remarkable inhibition of *E. coli* growth and proliferation was observed in solutions supplemented with heat-treated Reishi mushroom. The short-time heat treatment of the dry fruiting body of *G. lingzhi* revealed to be of significant applicability in the thermal processing of functional food and beverages without any negative attribution to its claimed medicinal and functional characteristics. However, it is noteworthy to mention that the final food matrix components of any newly developed products containing Reishi extract could interfere with the observed enhancements in our results if the final product were subjected to another round of thermal processing for the final packaging.

## Figures and Tables

**Table 1:** Effect of heat treatment on reducing sugars content (mean ± StDev) of Reishi mushroom (*G. lingzhi*).

Samples	Reducing Sugar Content (g 100 g^−1^)
Reishi Control	ND
Reishi 70 °C	ND
Reishi 120 °C	ND
Reishi 150 °C	0.230 ± 0.009 a
Reishi 180 °C	0.568 ± 0.012 b

Different letters are for statistically different values (Student’s *t*-test, *p* < 0.001). ND: not detected.

**Table 2:** The effect of heat-treated Reishi mushroom samples on the in vitro growth rate of *L. casei* and *E. coli* (mean ± StDev).

Heat-Treated Samples (20 min)	*E. coli* (Log CFU)		*L. casei* (Log CFU)	
	Initial Count	End Count	Initial Count	End Count
Reishi Control	7.075 ± 0.032 Ad	7.104 ± 0.027 Ac	4.719 ± 0.101 Aa	8.209 ± 0.017 Ba
Reishi 70 °C	7.055 ± 0.027 Acd	7.190 ± 0.126 Ac	5.556 ± 0.189 Ac	8.407 ± 0.025 Bb
Reishi 120 °C	7.004 ± 0.032 Aabc	6.802 ± 0.191 Ac	5.594 ± 0.027 Ac	9.334 ± 0.037 Bd
Reishi 150 °C	6.981 ± 0.025 Bab	5.358 ± 0.320 Ab	5.456 ± 0.023 Ac	8.730 ± 0.045 Bc
Reishi 180 °C	6.963 ± 0.014 Ba	2.774 ± 0.268 Aa	5.098 ± 0.069 Ab	8.085 ± 0.100 Bab
Frutafit HD inulin	7.034 ± 0.011 Abcd	7.033 ± 0.034 Ac	5.512 ± 0.088 Ac	9.252 ± 0.033 Bd

Upper case letters are for the time effect (Student’s paired *t*-test, *p* < 0.05) comparing the initial and end count; lower case letters show the temperature effect (Games-Howell’s post hoc test, *p* < 0.05) in columns. Initial count: after 24 h of culture; end count: after 72 h of culture. (*n* = 3 plates).

**Table 3:** Pearson correlation coefficients between the measured variables (upper triangle) with their significance levels (lower triangle).

	TEAC	DPPH	β-glucan	*E. coli* *	*E. coli* **	*L. casei* *	*L. casei* **	RS	TPC
TEAC	\	**0.958**	**0.911**	*−0.761*	*−0.557*	**0.571**	0.367	**0.533**	0.038
DPPH	**0.000**	\	**0.922**	*−0.794*	−0.482	**0.650**	**0.547**	0.444	0.100
β-glucan	**0.000**	**0.000**	\	*−0.826*	*−0.672*	**0.579**	0.291	**0.648**	−0.219
*E. coli* *	*0.001*	*0.000*	*0.000*	\	**0.748**	−0.327	−0.136	*−0.703*	0.284
*E. coli* **	*0.031*	0.069	*0.006*	**0.001**	\	0.151	0.393	*−0.991*	**0.772**
*L. casei* *	**0.026**	**0.009**	**0.024**	0.234	0.590	\	**0.665**	−0.172	0.480
*L. casei* **	0.178	**0.035**	0.292	0.628	0.147	**0.007**	\	−0.449	**0.724**
RS	**0.041**	0.098	**0.009**	*0.003*	*0.000*	0.539	0.093	\	*−0.793*
TPC	0.894	0.723	0.432	0.305	**0.001**	0.070	**0.002**	*0.000*	\

Significant positive correlations are in **bold**, significant negative correlations are written in *italics*, while insignificant correlations are written in grey. *: initial CFU count; **: end CFU count. TEAC: Trolox equivalent antioxidant capacity, DPPH: 2,2-diphenyl-1-picrylhidrazyl scavenging power, RS: Reducing Sugar Content, TPC: Total Phenolics Content.
